# Can pre-analytical procedures improve microbiological culture yield in patients with periprosthetic infections?

**DOI:** 10.1186/s12866-024-03493-0

**Published:** 2024-09-10

**Authors:** Juliane Käschner, Christoph Theil, Georg Gosheger, Jan Schwarze, Jan Pützler, Frieder Schaumburg, Burkhard Möllenbeck

**Affiliations:** 1 Department of General Orthopaedics and Tumor Orthopaedics, University Hospital Münster, Münster, Germany; 2 Institute of Medical Microbiology, University Hospital Münster, Münster, Germany

**Keywords:** Periprosthetic joint infection, Tissue culture, Revision arthroplasty

## Abstract

**Background:**

The detection of causative pathogens plays a crucial role in the diagnosis and targeted treatment of periprosthetic joint infections (PJI). While there have been improvements in analytic methods in the past, pre-analytical procedures have not yet been sufficiently investigated. The objective of this study was to compare the culture yield of four different pre-analytical procedures.

**Methods:**

Patients with perioperative diagnosis of PJI were included in a single center cross-sectional study (2021–2022). Tissue samples (*n* = 20) of each patient were randomly and equally distributed to each of the four study arms. Tissue samples were either send to the laboratory without culture medium (group A) or were transported in thioglycolate medium immediately after sampling at three different temperatures (room temperature, 4 °C, 37° for 24 h; group B-D). Culture media were investigated for growth on days 1, 3, 7, 12, 14. All organisms, the number of positive samples and the time to positivity were recorded and compared between the study arms. Single positive cultures were considered as contamination.

**Results:**

In total, 71 patients were included. The proportions of culture negative samples (10–15%) and polymicrobial infections (51–54%) were comparable between the four arms. Seven patients (10%) were culture-negative in group A, but showed growth in thioglycolate media (group B-D). Furthermore, 13% of patients showed growth in all groups, but additional organisms were cultured in thioglycolate. There was growth beyond day 7 of culturing only in thioglycolate, but not in group A. A storage temperature of 4 °C showed a longer time to positivity compared to the other groups (*p* < 0.001).

**Conclusions:**

Pre-analytical storage of tissue samples in thioglycolate broth did not improve the culture yield and did not detect additional cases of infection compared to the standard (pre-analytical storage in sterile containers). However, including a thioglycolate medium to the sampling algorithm reduced the rate of culture-negative infections and helped to identify additional organisms.

## Background

Periprosthetic joint infection (PJI) is one of the most common complications following hip or knee arthroplasties, reported with infection rates from 0.7–2.2% [1, 2]. Due to the annual increase in primary total hip arthroplasty (THA) and total knee arthroplasty (TKA), there has been a proportional increase in PJI [1, 3]. Furthermore, in patients who undergo revision arthroplasty, particularly in complex procedures with severe bone loss, the risk of PJI can exceed 10–25% [4]. The most common causative microorganisms of PJI are *Staphylococcus aureus* and coagulase-negative *Staphylococcus* species (CoNS) [2, 5]. Early and accurate diagnosis of PJI and the causative organism is important for an appropriate and successful treatment. Therefore, culture-negative infections are a challenge in the management of suspected PJI [6]. The proportion of culture-negative infections is approximately 6–22% of all PJIs [5–7], but can be as high as 42% [8]. Possible causes of culture-negative infections include antibiotic treatment prior to sampling, inadequate culture conditions (e.g. for the detection of bacteria in biofilms or fastidious species), short incubation times especially for low virulence infections, inadequate storage temperature, insufficient number of samples and prolonged transportation times [7, 9–20].

Despite advancements in diagnostic criteria over last years [21–24], tissue culture remains an important aspect in the diagnosis of PJI. To improve microbiological culture, various methods were developed to increase the culture yield such as the use of enrichment culture [11], tissue homogenization, manual milling or sonication [12], sonication of protheses [9, 10], next-generation sequencing of synovial fluid [25] or different PCR techniques [26, 27]. Nevertheless, the sensitivity, availability and costs of these methods is insufficient compared to tissue culture which therefore remains the gold standard in microbiological diagnosis. Specifically, one reason for reduced culture yield might be different demands of bacteria on their culture media. Thioglycolate broth is a non-selective medium that enables the growth of aerobic and anaerobic bacteria including fastidious species. It is therefore a candidate to improve the culture yield of infections with a high proportion of culture-negative samples [14]. Our hypothesis is that early culture in thioglycolate right after sampling may improve culture yield. Therefore, the purpose of this study was to identify the optimal pre-analytical temperature of inoculated thioglycolate broth before microbiological culture.

## Methods

### Ethics

Ethical approva was obtained by the local ethical committee (Ethikkommission der Ärztekammer Westfalen-Lippe, reference number 2019-708-f-S). All patients gave their written consent to participate in the study before any study related procedures.

### Study population

Patients were recruited at the orthopaedic department of University Hospital Muenster, Muenster, Germany between October 2021 and October 2022. They were included in a cross-sectional study, if the diagnosis of PJI (“confirmed” and “likely”) was established in accordance with the European Bone and Joint Infection Society (EBJIS) definition of periprosthetic joint infection [21, 22]. PJI was confirmed if at least one of the following criteria was present: a sinus tract communication with the prothesis or at least two positive separate fluid or tissue samples with the same microorganism. PJI was likely, when there was a clinical sign and laboratory or radiology sign found. These indicators included a serum C-reactive protein above 1 mg/dl, impaired wound healing, early radiographic loosening and synovial fluid white blood cell count above 1500/µl, synovial fluid polymorphonuclear neutrophil percentage above 65%, isolation of a microorganism in one tissue or fluid culture, positive histological analysis of periprosthetic tissue or presence of purulence in the affected joint. If PJI was unlikely, patients were excluded. All patients included in this study underwent staged surgical treatment for PJI and 12 weeks of systemic antibiotic treatment.

### Specimen collection

**Fig. 1:**
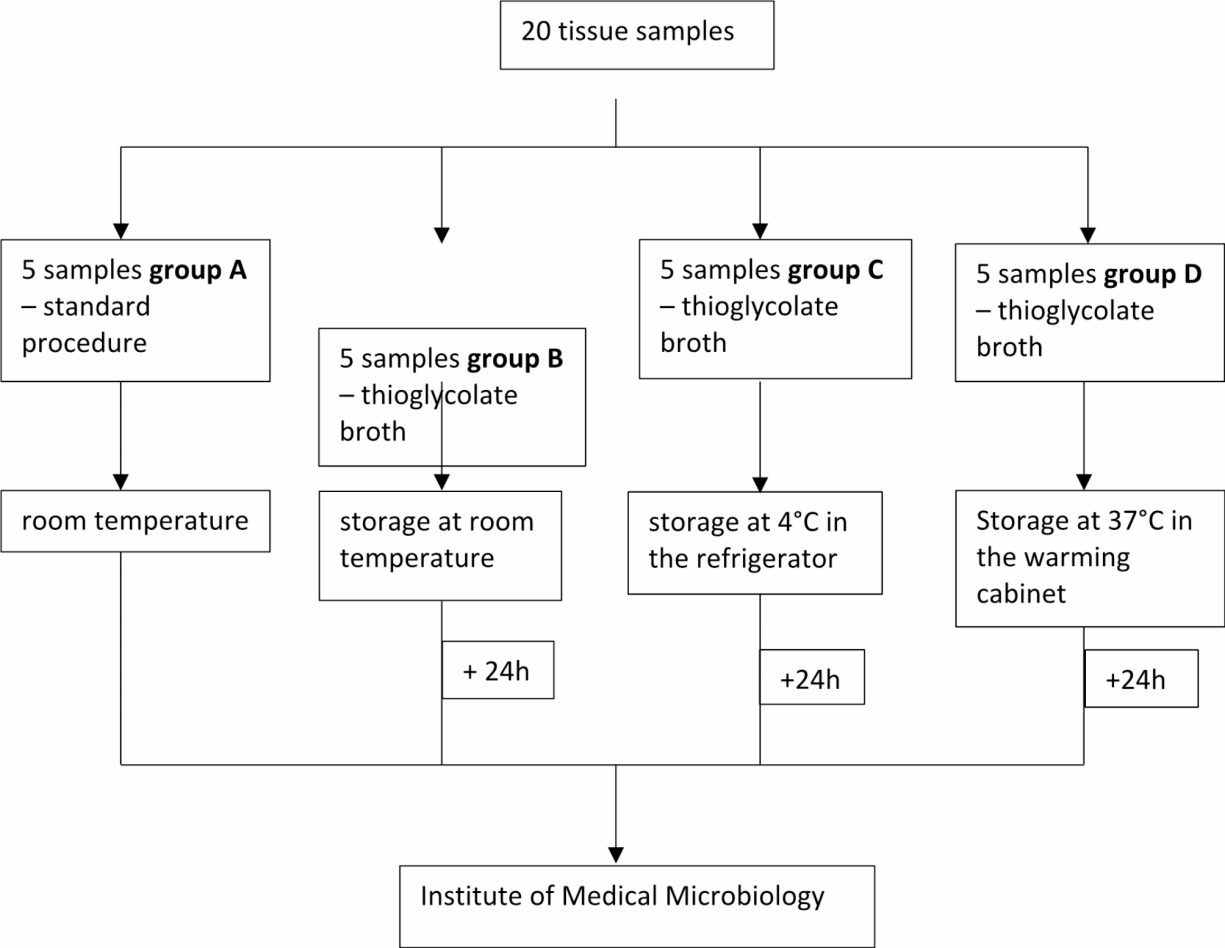
Pre-analytic algorithm Tissue samples (*n* = 20) of each patient were randomly and equally distributed to each study arm. They were sent to the laboratory without culture medium (group **A**) and were transported in thioglycolate medium after sampling at three different temperatures (room temperature, 4 °C, 37° for 24 h, group **B**-**D**)

Twenty tissue samples per patient were taken from five defined areas of the joint during surgery. These areas were selected because they appeared suspicious for infection, such as membranes, scar tissue, or bone. The samples were divided into four groups, with each group receiving five samples per patient (Fig. 1).

**Group A** represented the standard procedure. The samples were sent to the laboratory on the day of surgery in sterile plastic tubes without any culture medium and kept at room temperature. In **groups B – D**, the samples were placed in thioglycolate broth, along with Hemine and Vitamin K1 (Oxoid, Wesel, Germany), during surgery. These samples were stored in the operating room (OR) for 24 h (to simulate long transportation times) and transported to the laboratory on day 1 after surgery. The samples of **group B** were kept at room temperature, while those in **group C** were stored in a refrigerator at 4 °C and those of **group D** were placed in an incubator at 37 °C before being sent to the lab.

### Conventional microbiologic methods

Samples of group A were homogenized with a sterile scalpel and inoculated in thioglycolate in the laboratory (Fig. 1). Samples of the other arms were left in the thioglycolate broth. For each sample 10 µl of thioglycolate broth per plate or an equivalent volume of the tissue of group A were then cultured on different types of agar plates. We incubated the samples on Columbia blood agar for two days in ambient air, on chocolate agar for two days in a 5% carbon dioxide environment and on Schaedler agar for three days under anaerobic conditions (all plates purchased from BD, Heidelberg, Germany). All plates were incubated at 37 °C. On day 3, 7, 14 after sampling, enrichment thioglycolate broths were subcultured on chocolate agar and Schaedler agar, both under the above conditions. The cultures were examined daily for possible microbial growth. Species identification was done with MALDI-TOF mass spectrometry (Bruker, Bremen, Germany).

To meet the criteria of infection, at least 2 out of 5 samples per arm had to be positive for the same organism [28]. Single positive cultures were considered as contamination.

### Statistical analysis

All grown organisms, the different species, the time to positivity, pre-operative aspiration results, the number of positive samples, polymicrobial samples and contamination were compared by using descriptive statistics. Groups were compared using Cochran-Q-Test and Friedman´s test. A P value < 0.05 was considered significant. All analysis were performed using IBM SPSS statistics version 29.

## Results

### Microbiology findings

The final dataset included 71 patients (40 knee and 31 hip PJIs). The median age of the included 31 male and 40 female patients was 71 years. 22% patients received preoperative antibiotic therapy (16/71). All characteristics of the patients are illustrated in Table 1.

**Table 1:** Characteristics of the patients

Characteristics	Patients
Age - yr	
Median	71
Range	36–88
Sex – no. (%)	
Male	31 (44)
Female	40 (56)
Site of arthroplasty – no. (%)	
Knee	40 (56)
Hip	31 (44)
Preoperative laboratory findings – no./total no. (%)	
Blood leukocyte count > 10 × 10^9^/liter ⸯ	17/70 (24)*
Serum C-reactive protein > 1 mg/dl	47/71 (66)
Synovial-fluid leukocyte count > 1500/µl	33/38 (87)
Synovial-fluid differential > 65% neutrophils	26/32 (81)
Preoperative antibiotics – no. (%)	16 (22)
Diagnostic criterion for PJI	
Sinus tract – no. (%)	22 (31)
Purulence – no. (%)	55 (77)
Acute inflammation in tissue – no./total no. (%)	32/58 (55)

*Since most patients had a chronic infection, inflammation values were mostly not elevated

Coagulase-negative staphylococci were detected in the majority of patients (depending on the study arm: 15–18 patients) followed by *Streptococcus agalactiae* (4–5 patients, Table 2). There were no significant differences in the rate of culture-negativity (*p* = 0.825) or polymicrobial growth (*p* = 0.147) between the standard procedure and each thioglycolate group. In particular, culture yield was comparable between all four study arms.

**Table 2:** Microbiological findings in standard und thioglycolate groups

	Study arm			
Species	A	B	C	D
Coagulase-negative *Staphylococcus* spp.	18	16	15	17
*S. epidermidis*	6	5	6	8
*S. caprae*	4	4	4	4
*S. haemolyticus*		1		
*S. capitis*	3	2	2	2
*S. hominis*			1	
*S. lugdunensis*	3	2	1	2
Other coagulase-negative Staphylococci	2	1	1	1
*Staphylococcus aureus*	2	4	3	3
Viridans-streptococci	1	1	1	1
*Streptococcus agalactiae*	4	4	5	4
*Granulicatella adiacens*	1	1	1	1
*Entereococcus* spp.	2	3	3	2
*Escherichia coli*	1	2	2	1
*Enterobacter* spp.	2	1	1	2
*Pseudomonas* spp.	3	3	3	4
*Proteus mirabilis*				1
*Klebsiella* spp.	1	1	1	1
*Serratia marcescens*	1	1	1	1
*Achromobacter* spp.				1
*Cutibacterium* spp.	3	3	4	3
*Dermabacter hominis*	1	1	1	1
*Aerococcaceae* spp.	1		1	
*Micrococcus* spp.				1
*Anaerococcus* spp.			1	
*Corynebacterium* spp.	1	1	1	1
*Bacillus* spp.		1		
*Facklamia* sp.				1
Monomicrobial	26 (37%)	26 (37%)	29 (41%)	22 (31%)
Polymicrobial	7 (10%)	7 (10%)	6 (8%)	11 (15%)
Culture-negative	38 (54%)	38 (54%)	36 (51%)	38 (54%)
Single-positive culture	19 (27%)	12 (17%)	10 (14%)	10 (14%)

The sum of all individual results does not correspond to the sum of the patients, as some patients showed polymicrobial results

The most common cultured microorganisms were coagulase-negative *Staphylococcus* species (CoNS), *Streptococcus agalactiae*,* Cutibacterium* species and *Staphylococcus aureus* (Table 2). There were no differences in culture positivity for individual species.

A total of 10% (7/71) of patients would have remained culture-negative in the standard culture, but cultures were positive in one or more of the thioglycolate groups (Table 3). In particular, one case of a PJI caused by *Cutibacterium* spp. was only identified in thioglycolate cultures. The other patients who were only positive in the thioglycolate groups showed growth of *Bacillus spp.*,* E. coli*,* Enterococcus* spp., *Cutibacterium* spp. and CoNS each.

**Table 3:** Detected species in the different study arms

		Study arm (No. of patients)			
		A	B	C	D
	No. of patients	Standard	Thioglycolate at room temperature	Organism in thioglycolate at 4 °C	Organism in thioglycolate at 37 °C
Total	71				
Positive thioglycolate culture (at least one group) and negative standard culture	7	- - - - -	- *Bacillus spp.* (1) *Escherichia coli* + *Enterococcus* spp. (1) *Cutibacterium* spp. (1) CoNS* (1)	- - *Escherichia coli* + *Enterococcus* spp. (1) *Cutibacterium* spp. (2)	*Achromobacter* spp. (1) - - *Cutibacterium* spp. (1) CoNS* (1)
Positive thioglycolate (at least one group) and standard culture	33				
Concordant	21	CoNS* (11) *Enterococcus* spp. (1) *Pseudomonas* spp. (2) *S. agalactiae* (2) *S. aureus* (1) *Granulicatella* spp. *+* CoNS* (1) *Viridans - streptococci* (1) *Aerococcus* spp. (1) *Enterococcus* spp. + *Corynebacterium* spp. (1)	CoNS* (10) *Enterococcus* spp. (1) *Pseudomonas* spp. (2) *S. agalactiae* (2) *S. aureus* (1) *Granulicatella* spp. *+* CoNS* (1) *Viridans – streptococci* (1) - *Enterococcus* spp. + *Corynebacterium* spp. (1)	CoNS* (10) *Enterococcus* spp. (1) *Pseudomonas* spp. (2) *S. agalactiae* (2) *S. aureus* (1) *Granulicatella* spp. (1) *Viridans – streptococci* (1) *Aerococcus* spp. (1) *Enterococcus* spp. *+ Corynebacterium* spp. (1)	CoNS* (10) *Enterococcus* spp. (1) *Pseudomonas* spp. (2) *S. agalactiae* (2) *S. aureus* (1) *Granulicatella* spp. *+* CoNS* (1) *Viridans – streptococci* (1) - *Enterococcus* spp. + *Corynebacterium* spp. (1)
Discordant	12				
Additional organism detected in thioglycolate culture	9	CoNS*+ S. agalactiae (1) CoNS* (1) CoNS* (1) *S. agalactiae* (1) *Escherichia coli* (1) *Pseudomonas* spp. (1) *Enterobacter* spp. (1) *Enterobacter* spp. + *Dermobacte hominis + Klebsiella* spp. (1) *S. aureus + Serratia* spp. (1)	CoNS*+ S. agalactiae + S. aureus (1) CoNS* (1) CoNS* (1) *S. agalactiae* + *S. aureus* (1) *Escherichia coli* (1) *Pseudomonas* spp. (1) - *Enterobacter* spp. + *Dermobacter hominis* + *Klebsiella* spp. *+* CoNS* + *Facklamia* spp. (1) *S. aureus + Serratia* spp. (1)	CoNS*+ S. agalactiae (1) CoNS* + S. aureus (1) CoNS* (1) *S. agalactiae* (1) *Escherichia coli* (1) *Pseudomonas* spp. (1) CoNS* (1) *Enterobacter spp. + Dermobacter hominis + Klebsiella* spp. *+ Anaerococcus* spp. (1) *S. aureus + Serratia* spp. *+ Pseudomonas* spp. (1)	CoNS*+ S. agalactiae (1) CoNS* (1) CoNS* + Proteus mirabilis (1) *S. agalactiae + S. aureus* (1) *Escherichia coli +* CoNS* (1) *Pseudomonas* spp. *+* CoNS* (1) *Enterobacter* spp. (1) *Enterobacter* spp. + *Dermobacter hominis.* + *Klebsiella* spp. (1) *S. aureus + Serratia* spp. *+ Pseudomonas* spp. (1)
Additional organism detected in standard culture	2	*Cutibacterium* spp. + CoNS* (2)	*Cutibacterium* spp. (2)	*Cutibacterium* spp. (2)	*Cutibacterium* spp. (2)
Different organisms detected	1	*Cutibacterium* spp. (1)		CoNS* (1)	
Negative thioglycolate culture and positive standard culture	0				
Negative thioglycolate and standard culture	31				

*CoNS – coagulase – negative *Staphylococcus* species

In contrast, no patient who was culture-negative in thioglycolate cultures showed bacterial growth in the standard culture.

Nonetheless, 44% (31/71) of patients were culture negative in both, standard procedure and thioglycolate groups.

The remaining 46% (33/71) had bacterial detection in both, standard group and at least one thioglycolate group, with 21 cases of concordant growth and 12 cases of discordant growth (Table 3). In nine cases (13%) of discordant culture results, additional, new microorganisms could be detected in the thioglycolate groups, three times *S. aureus*. In contrast, in two cases there was additional pathogen detection by the standard method (CoNS).

In terms of single positive tissue culture, a significant difference between all four groups could be demonstrated, with a higher rate in the standard group (*p* = 0.044).

Preoperative antibiotic therapy was not associated with negative cultures throughout the different study groups (group A: *p* = 0.407, group B: *p* = 1, group C: *p* = 0.267, group D: *p* = 1).

The time to positivity was significantly different between the groups (*p* < 0.001), with a slower growth in thioglycolate group at 4 °C. The median number of days until the first positive culture was one day for the standard group (range: 1–7 days), thioglycolate group at room temperature (range: 1–14 days) and thioglycolate group at 37 °C (range: 1–14 days). For thioglycolate group at 4 °C the median number of days until the first positive culture were two days (range: 1–14). Five cases of microbial detection (CoNS, *Cutibacterium* spp., *Enterobacter* spp.) in thioglycolate groups would have been missed, if the cultivation time would have been set at 7 days instead of 14 days.

## Discussion

This study found that the use of thioglycolate as a transportation and culture medium led to additional positive cultures in 10% of patients who otherwise would have been culture–negative if transported in sterile containers without a medium. Furthermore, in 13% of patients additional organisms were found, who would have been missed with standard culture. On the other hand, culture storage temperature during the first 24 h after sampling had no effect on culture yield or time to positive culture. Consequently, we recommend the use of thioglycolate as an additional culture medium during surgery for PJI in order to optimize culture yield.

There is a lack of evidence for the selection of the optimal culture medium for microbiological culture in PJI. We found that the use of thioglycolate broth directly in the operation room was suitable for isolating both, aerobic and anaerobic microorganisms, with a median time to positivity of one day for storage temperature at 37 °C and room temperature and two days for 4 °C. In particular, high-virulent organisms, missed in standard procedure, could be detected in thioglycolate groups (Table 3). Our results support the findings of Bossard et al. [14]. They exclusively investigated the effect of thioglycolate on the detection of *Cutibacterium acnes* in patients undergoing orthopaedic surgery for bone or joint infection, including 35 cases of PJI. The samples were incubated for up to ten days on anaerobic or aerobic agar plates and in aerobic thioglycolate broth. They concluded that thioglycolate had a higher sensitivity in detecting *Cutibacterium acnes* in PJI than culture media for aerobic and anaerobic agar plates (66.3% vs. 5.1% and 42.1%). The median time to positivity for all methods was six days. A retrospective study by Rieber et al. [16] also showed a higher microbial detection rate in thioglycolate broth. They investigated the amount and time to positivity of microbial growth in 25 cases of anaerobic-induced PJI by incubating anaerobically cultured plates, brain-heart infusion broth and liver thioglycolate broth for 14 days. Bacterial growth of anaerobes was first detected in thioglycolate broth in all 25 cases within six days. In the brain-heart infusion broth and anaerobic culture plates, microbial growth was detected in 11 and 18 cases, respectively, after prolonged incubation, demonstrating the need to use the most appropriate culture medium to improve the quality of PJI diagnosis. The faster detection of anaerobic microorganisms in thioglycolate broth, so that prolonged incubation would not be necessary, contrasts with our results.

The time to positivity in our study was one to 14 days for all thioglycolate groups. If the cultivation period had been limited to seven days, five microorganisms would have been missed in thioglycolate groups (CoNS, *Enterobacter* spp., *Bacillus* spp.). For the standard procedure, seven days appears to be sufficient to detect most organisms and keep the risk of contamination as low as possible. Concerning duration of cultivation, there is a controversial discussion in literature without a defined optimal standard [24]. An incubation time of five to seven days is most common. Klement et al. [29] found no differences in culture yield between a cultivation duration of five days or 14 days in a study on 189 patients with PJI. On the other hand, Tarabichi et al. [18], Talsma et al. [19] and Birlutiu et al. [20] recommend a prolonged cultivating time for 10–14 days to find the majority of organisms, because of different time to positivity for different species. Talsma et al. [19] and Birlutiu et al. [20] emphasize in particular a difference in the time to positivity between low and high virulent pathogens. Tarabichi et al. [18] investigated aerobic, anaerobic, acid-fast bacilli and fungal cultures of 536 patients. Aerobic and anaerobic cultures were done for 14 days, and fungal cultures for a minimum of 21 days. The mean time to positivity (TTP) for all positive cultures was 3.3 days. TTP between the different microorganisms and sample types was significant different, gram-negative organisms were isolated significantly faster (1.99 days) than gram-positive organisms (3.33 days, *p* < 0.001) [18]. Considering that their study included a greater number of samples, it is possible that with our study design differences in TTP between species were missed but should still be considered in clinical practice. Bossard et al. [14] also advocate an incubation period longer than 7 days despite the increasing risk of cultivating bacterial contamination at extended culture duration. A total of 21.4% of infections would have been missed in their study if incubation had lasted only 7 days. They recommend an extended incubation period of up to 10 days and a blind subculture in thioglycolate broth after 10 days in cases with a high suspicion of *C. acnes* infection. In this study, 8.6% of cases would have been missed if cultures had been stopped on day 10 [14]. In our study, the detection of bacteria with reduced growth rate or intracellular persistence in the thioglycolate approaches may be a reason for the wider range of time to positivity in the thioglycolate groups.

In the present study, storage temperature during the first 24 h had no impact on culture results although a slower growth was found in the group that was stored at 4 °C. This approach was chosen in order to simulate prolonged transportation time that may occur in real life scenarios where a microbiology lab is not close by or promptly available. Whereas Van Cauter et al. [13] demonstrated significantly higher culture yield of *S. epidermidis* when samples that were stored at 4 °C, when cultivation within two hours as recommended in the Infection Diseases Society of America (IDSA) guidline [30] was not possible. In their study, they incubated *S. epidermidis* in femoral heads removed during hip arthroplasty. Considering that their study focused on *S. epidermidis*, it is possible that storage temperature might have an effect on culture yield for this bacterial species, but the general effect might be small as our study including aerobic and anaerobic, gram-positive and negative species was unable to detect an effect. Still transportation delays are common as Blevins et al. [17] reported that even in the best-case scenario, delays in vivo occur for a variety of reasons making it difficult to process samples within two hours even in specialized centres with microbiology labs on the same premises.

We detected a significant higher rate of single positive cultures, which were considered as contamination, in standard procedure than in thioglycolate groups. Considering the dilemma that single positive cultures pose for surgeons and infectious diseases physicians in patients with preoperative diagnosis of infection, sample collection and processing must be optimized as well. Careful preparation of tissue samples, such as dividing the samples with scalpels, pestles or mortars, as well as semi-automated homogenization, which allows the best release of bacteria with less contamination than manual methods should be standard of care and is usually performed in our department. However, as part of the study, homogenisation for the samples from thioglycolate arms (group B–D) was technically not possible as tissue samples were already placed in the broth before shipment. Therefore, considering our results, that actual benefit might be more considerable if optimized tissue homogenisation could be used for these samples as well [12].

While this study investigates a novel approach in culture preparation, it is limited by several factors that need to be considered when interpreting the results presented: considering that this is a pilot study with an unknown effect size of the thioglycolate cultures, funding and ethical approval was secured for one year resulting in the presented number of patients included. However, the number of patients with individual organisms available for comparison might be too small to achieve statistically significant results. For future studies, a greater number of patients should be included. Secondly, there is a high rate of culture-negative infections throughout all groups. We attribute this to the fact that many patients were transferred to our department after an initial washout and debridement procedure at an outside hospital that failed to undergo definitive surgical treatment. Therefore, there might have been organisms cultured before at the time of the procedure at an outside institution, but not at our hospital as part of the study protocol. Furthermore, 22% of patients had undergone recent antibiotic treatment although in this study there was no statistical impact on culture negativity. Additionally, several organisms cultured are considered rare in patients with PJI, but were included as part of the study’s algorithm. Molecular methods could be carried out in addition to the tissue samples to prove whether the pathogens are the causative pathogens or a potential contamination.

In conclusion, we found using thioglycolate as culture medium did not increase the overall culture yield but detected additional microorganisms and reduced the risk of culture negativity. While storage temperature did not influence culture results, additional growth after one week was found only in thioglycolate culture.

## Data Availability

The datasets used and/or analyzed during the current study are available from the corresponding author upon reasonable request.
